# Antifungal and Coagulation Properties of a Copper (I) Oxide Nanopowder Produced by Out-of-Phase Pulsed Sonoelectrochemistry

**DOI:** 10.3390/antibiotics13030286

**Published:** 2024-03-21

**Authors:** Valérie Mancier, Sirine Fattoum, Hélène Haguet, Julie Laloy, Christina Maillet, Sophie C. Gangloff, Jean-Paul Chopart

**Affiliations:** 1 Université de Reims Champagne-Ardenne (URCA), Institut de Thermique, Mécanique et Matériaux (ITheMM, UR 7548), BP 1039, 51687 Reims, France; sirine.fattoum@etudiant.univ-reims.fr; 2 Université de Reims Champagne-Ardenne (URCA), MATériaux et Ingénierie Mécanique (MATIM, UR 3689), BP 1039, 51687 Reims, France; jean-paul.chopart@univ-reims.fr; 3 Département de Pharmacie, University of Namur (UNamur), Rue de Bruxelles 61, 5000 Namur, Belgium; 4 Université de Reims Champagne-Ardenne (URCA), Biomatériaux et Inflammation en Site Osseux (BIOS), 51097 Reims, France; christina.maillet@univ-reims.fr

**Keywords:** copper (I) oxide, cuprite, nanoparticles, sonoelectrochemistry, antifungal materials, *Candida albicans*, platelet, hemostasis

## Abstract

Copper (I) oxide (cuprite) is a material widely used nowadays, and its versatility is further amplified when it is brought to the nanometric size. Among the possible applications of this nanomaterial, one of the most interesting is that in the medical field. This paper presents a cuprite nanopowder study with the aim of employing it in medical applications. With regards to the environmental context, the synthesis used is related to green chemistry since the technique (out-of-phase pulsed electrochemistry) uses few chemical products via electricity consumption and soft conditions of temperature and pressure. After different physico-chemical characterizations, the nanopowder was tested on the *Candida albicans* to determine its fungicide activity and on human blood to estimate its hemocompatibility. The results show that 2 mg of this nanopowder diluted in 30 µL Sabouraud broth was able to react with *Candida albicans*. The hemocompatibility tests indicate that for 25 to 100 µg/mL of nanopowder in an aqueous medium, the powder was not toxic for human blood (no hemolysis nor platelet aggregation) but promoted blood coagulation. It appears, therefore, as a potential candidate for the functionalization of matrices for medical applications (wound dressing or operating field, for example).

## 1. Introduction

Nanoparticles (NPcs) are currently largely employed in various fields such as food (additives), environment (sensor or low carbon energy production), and medicine. In this last item, the NPcs can be employed to deliver drugs optimally in the whole body [1] or be employed as contrast agents but also for microbiocide reasons. Metals (such as silver or copper) or metallic oxides (zinc oxide, copper oxides, etc.) can be used for these applications because they are efficient in killing common bacteria such as *Escherichia coli* (*E. coli*), methicillin-resistant *Staphylococcus aureus* (MRSA), or *Pseudomonas aeruginosa* [2]. Some of them are, therefore, already included in textiles (silver NPcs in socks and wound dressings, for example), and more research on this item is already ongoing [3]. The same materials are also often efficient against fungi such as *Alternaria alternata*, *Botrytis cinerea*, *Monilia fructicola* [4], *Saccharomyces cerevisiae* [5], and *Candida albicans* (*C. albicans*) [6,7,8]. Whatever their chemical nature, NPcs are, however, susceptible to impairing human safety due to their nanometric size, which makes them highly biologically active. They can, therefore, disrupt normal biological mechanisms and lead to systemic or organic pathologies [9]. In addition, their nanometric size allows them to easily pass cell membranes and enter the systemic circulation, inducing an important dissemination throughout the body. Whatever their route of exposure, nanomaterials can enter the bloodstream, where they can interact with various blood cells [10], making the assessment of hemocompatibility (i.e., the interaction between nanomaterials and blood components) an essential step, independently of the application type [11]. Hemocompatibility testing focuses on assessing the impact of NPcs on red blood cells and platelets, whereas few studies assess the impact on blood coagulation. However, when nanoparticles encounter blood, plasma proteins instantly adsorb to the surface of NPcs to form a protein corona that may influence their activity, potentially impacting the activity of coagulation factors [9], and more and more papers encourage researchers to evaluate the interactions of NPcs with all the constituents of blood [9].

This paper is a complement to already published works [12,13] concerning the synthesis of copper (I) oxide nanopowder (NPw) and the physical characterizations and antibacterial properties of the raw NPcs obtained. This oxide was initially chosen because this material is less studied than metals or other metallic oxides and because, even if Cu_2_O has already been classified as a toxic agent for aquatic animals [14,15], its toxicity is nonetheless contested for mammals [16,17], unlike CuO. A supplementary advantage of copper (I) oxide for medical applications is also that it is a mineral compound that can helpfully replace organic molecules. Indeed, it will potentially allow an increased therapeutic arsenal because only four main classes of antimycotic drugs are currently available with low secondary effects, and the increased resistance of fungi to drugs is a reality [14,18]. Moreover, it is versatile and, therefore, employable in different application fields in addition to the health field that is focused on in this work. This point also explains why copper (I) oxide was preferred to another mineral bactericide compound such as silver. Indeed, silver is a more expensive metal, and its ions are classified as toxic for humans [19]. It is also much less versatile than copper oxides. Our choice is also based on the possibility of elaborating them by out-of-phase pulsed sonoelectrochemistry, which is an uncommon technique. Indeed, many more classical ways exist to produce nanomaterials, such as mechanical erosion, laser ablation, or thermal decomposition [20,21]. Why, therefore, choose the out-of-phase pulsed sonoelectrochemistry? The aim was to be in accordance with the “One health” concept because this technique allows limited chemical product consumption and the use of an “environmentally friendly” technique. Moreover, there is a valid possibility of industrial-scale applications [22]. From a morphological point of view, this method is also chosen because it produces particular NPcs since they are faceted. This characteristic influences their antibacterial properties [23]; thus, it is interesting to research if the fungicide behavior is modified too.

The first part of this work was previously published [13] to exhibit experimental details of the copper (I) oxide NPw elaboration and the results of the main physico-chemical analyses and the antibacterial tests. They show that the sonoelectrochemical powder is efficient in small quantities (2 mg diluted in 30 µL trypto casein soy) against MRSA and *E. coli*. This article exhibits the deepening of some physico-chemical analyses and a focus on fungicide activity as well as hemocompatibility for this same material. The choice was made to study *C. albicans* because it is a commensal fungus (gastric, respiratory, and genital tracts) often responsible via its toxin for many nosocomial diseases [24] and because of its possibility to form biofilms that are more tolerant to molecules [25]. Cu_2_O NPcs could, therefore, be inserted into operative sheets in hospitals and wound dressings and could offer both fungicide and bactericide effects if the quantity of NPcs needed is of the same order. Since injuries promote the transfer of micro-organisms to blood circulation, the hemocompatibility of NPcs also needed to be investigated. Prior studies assess the impact of copper-containing NPcs on hemolysis but present discordant results [26,27,28,29]. Indeed, the toxicity of copper-containing nanomaterial towards red blood cells is dependent on numerous factors, including size, shape, porosity, surface area, and chemical composition, making it hardly predictable and making comparisons between studies hardly possible [28]. However, this estimation is essential as a first step to estimate the real potential medical applications.

## 2. Results and Discussion

### 2.1. Nanopowder Characterization

All the studied NPws exhibit X-ray diffraction patterns similar to those already published and extensively analyzed in detail [13]. Once again, it has been confirmed that sonoelectrochemical NPcs are consistent with the expected cubic crystallographic structure and that they are made of pure copper oxide (I), free from impurities such as copper oxide (II) CuO and from titanium horn in particular.

#### 2.1.1. TEM Analysis

**Figure 1:**
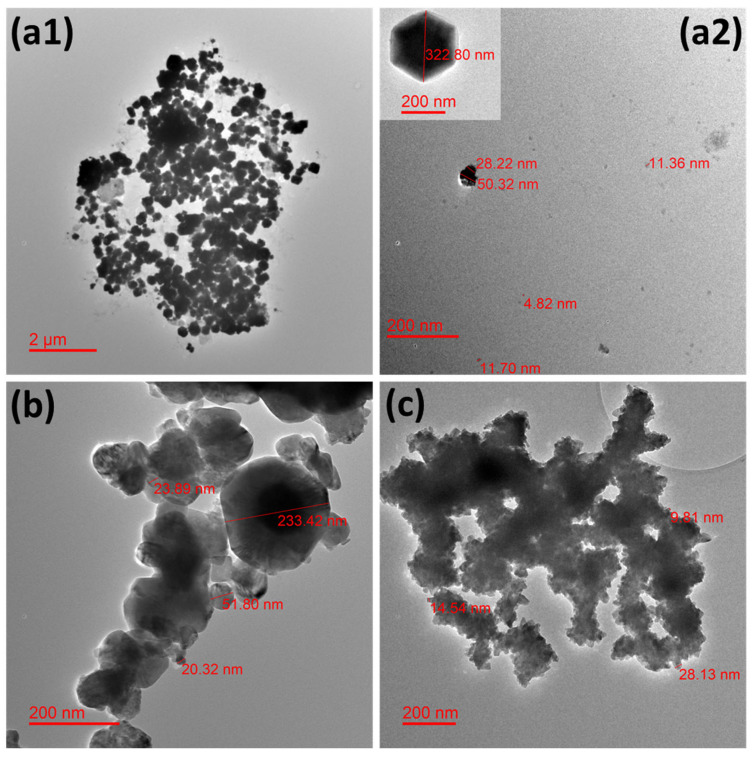
Transmission electron microphotographs showing the morphology of the different studied nanopowders. Micrographs for (**a1**,**a2**) electrochemical synthesized cuprite nanopowder, (**b**) commercial cuprite nanopowder, and (**c**) commercial copper nanopowder.

A few representative TEM microphotographs are exhibited in Figure 1 for all the studied NPws. Sonoelectrochemical copper oxide powder constitutes elements of diverse sizes with a global trend to agglomerate (Figure 1(a1)). Particles with particular shapes (hexagonal and cubic (Figure 1(a2))) are found in accordance with our previous work [13].

Such geometrical shapes would likely come from the technique used in which small germs of oxide are electrodeposited in a first step on the titanium sonotrode surface. Indeed, electrodeposition is easier on cuprite when some germs of this material are synthesized because the involved materials are identical. It is also possible that not all oxide germs are expelled from this same surface during the following ultrasound pulse because the level of ultrasound power was chosen to avoid the erosion of the sonotrode and, therefore, to avoid contaminating the powder with titanium. In this case, germs could grow and lead to the biggest NPcs observed. However, small free and small agglomerates of NPcs are also visible (Figure 1(a2)). The dispersion of NPcs could be improved if a surfactant such as sodium dodecyl sulfate [30] or cetyltrimethylammonium [31] was employed during the NPw synthesis, but the choice was to employ the raw NPcs in the first experiments, mainly so as not to disturb antimicrobial results as well as hemocompatibility tests. For the commercial copper oxide NPw, single particles are also difficult to observe, and polydispersion of sizes is clearly shown in Figure 1b. Commercial copper seems more homogenous from a granulometric point of view, but a strong agglomeration of the particles is obvious in Figure 1c. It is, therefore, important to remember that the TEM grid deposition process undoubtedly promotes cluster formation via the evaporation of ethanol-containing powder droplets.

#### 2.1.2. Particle Size Measurements

**Figure 2:**
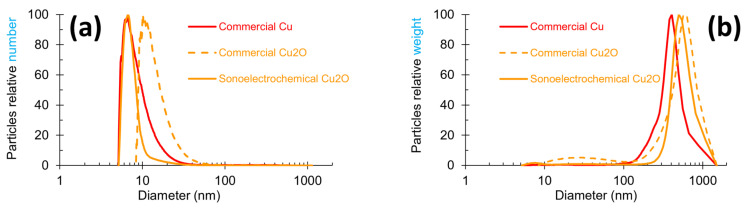
Particle size profiles obtained by centrifugal liquid sedimentation at 22,000 rpm in two modes for the three studied nanopowders: (**a**) relative number and (**b**) relative weight mode for sonoelectrochemical Cu_2_O, commercial Cu_2_O, and commercial Cu nanopowders.

Particle size measurements were carried out on all the NPws, and the graphic distribution of the NPcs sizes in each of them is exhibited in Figure 2 with two modes of representation: number and weight. The number mode focuses on the most numerous particles, while the weight mode targets the heaviest particles. These two modes can, therefore, give complementary data. A global observation of Figure 2 shows that the shape of the three powders’ profiles slightly differ in the width of the peak and the maximum peak value. In Figure 2a, commercial copper and sonoelectrochemical oxide copper overlap in the range of 5–8 nm, and the peak width is clearly smaller for the laboratory oxide copper. The peak profile of commercial cuprite has a shape similar to the copper one, but it is shifted towards a higher NPcs diameter range (8 to 50 nm). Figure 2b shows that besides the nanometric particles previously observed, a population of particles with submicronic diameters is present. These particles are less numerous (no peak visible in the number mode graph (Figure 2a) for diameters superior to 100 nm), but they are very heavy in the three samples. The diameter of these bigger particles exceeds roughly 100 nm for all the powders.

A peak width between 10 and 100 nm can be observed for commercial Cu_2_O NPw, but referring to the scale of the graph, the NPcs of this diameter range are negligible in the whole mass of the powder. All these data are coherent with the maximum peak values reported in Table 1.

Indeed, this table shows clearly that ultrafine particles exist inside all NPws (the value of the maximum peaks corresponding to a maximum particle diameter of 10 nm in number mode) but that bigger particles with a diameter greater than 400 nm are also included. These points can be linked to TEM results.

An in-depth study of the different diameter ranges has led to a better estimate of the quality of the materials and the relevance of comparisons for the next studies. The different data extracted are registered in Table 2 and Table 3.

The number mode graph data (Table 2) revealed that the finest powder is the sonoelectrochemical Cu_2_O one, with 79% of particles less than 10 nm. Opposing this, the less fine NPw is the commercial Cu one, with 14% of particles exceeding 100 nm, i.e., about seven times more than the two Cu_2_O NPws. However, the size repartition for this material is consistent with the datum supplied by the manufacturer (84% of the NPcs’ sizes are smaller than 50 nm when this product is guaranteed to contain at least 50% NPcs of 50 nm diameter).

Additional information was extracted from the data in the relative weight graph (Table 3). Indeed, the Cu_2_O NPw was found to be more heterogenous than the Cu NPw since, in addition to their ultrafine particles, around 75% of the mass of the material came from elements (unique particles or aggregates of particles) sizing more than 500 nm (compared to 33% for copper). This point is supported by the TEM microphotographs. However, the mass percentages in the three NPws are similar (mean value equal to 97%) when the particle size exceeds 250 nm: 100%, 97%, and 94% for sonoelectrochemical copper oxide, commercial copper oxide, and commercial copper NPws, respectively.

Furthermore, these results are very similar to those already published in our previous paper [13].

#### 2.1.3. Specific Area Determination

**Figure 3:**
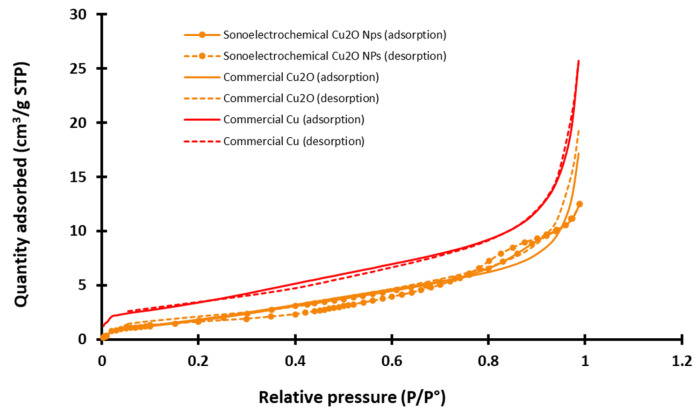
Example of adsorption/desorption azote isotherms graphs recorded for the three studied nanopowders (sonoelectrochemical and commercial copper (I) oxide and for commercial copper) (STP = standard temperature and pressure, P/P° = relative pressure of nitrogen).

A Brunauer–Emmet–Teller analysis was performed to access the specific area that is an important parameter to better compare the potential reactivity of the three NPws and their potential effect on hemotoxicity [32,33]. An example of nitrogen adsorption/desorption azote isotherm graphs recorded at −196 °C for all NPws is exhibited in Figure 3.

Experimental isotherms for the two commercial NPws are nearly reversible, and according to the classification of the International Union of Pure and Applied Chemistry (IUPAC) that distinguishes six different shapes of physisorption [34], they can be classified as type II. It is more complex for the sonoelectrochemical NPw. Indeed, the isotherms have two features of type IV since they exhibit a hysteresis and two inflection points, but the theoretical final saturation of quantity adsorbed is not visible after the hysteresis. Moreover, the adsorption and desorption curves intersect. The type of hysteresis loop is, therefore, uncommon and cannot be identified using the IUPAC classification. This point could be explained by the small quantity of analyzed material, but the exploitation of the graphs makes it possible to extract the specific area value. Therefore, the found value is around 5 and 6 m^2^·g^−1^ for sonoelectrochemical and commercial Cu_2_O, validating the relevance of comparing these two chemical natures of the NPws. With a specific area only double for the copper NPw (12 m^2^·g^−1^), the use of this material as a positive standard in antifungal tests also appears relevant.

### 2.2. Antifungal Activities of Nanopowders

**Figure 4:**
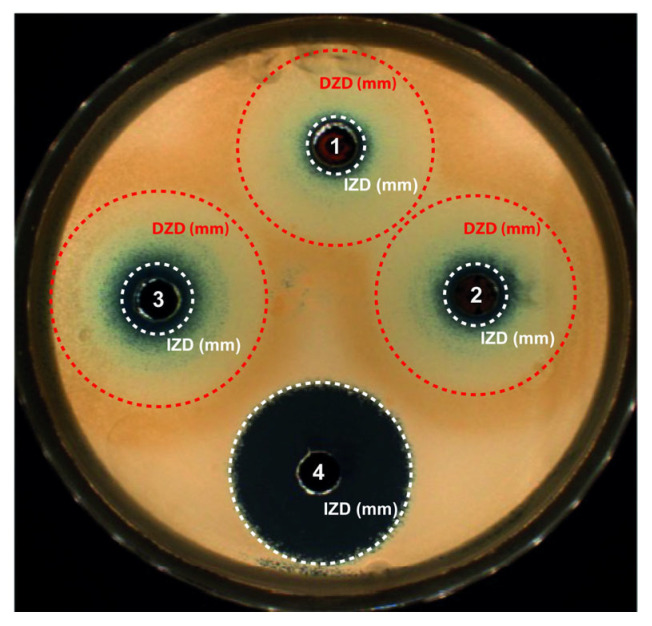
Antifungal activity after 24 h of incubation at 37 °C and exposure to the different nanopowders on Sabouraud dextrose agar seeded with *Candida albicans*. Results for 2 mg of nanopowder placed in a well and diluted in 30 µL of Sabouraud medium: (1) Cu_2_O, (2) commercial Cu_2_O or (3) commercial Cu nanopowder, and (4) bleach used as positive control. Circles around the wells report the growth inhibition zone diameter (white circle) and the growth disturbed zone diameter (red circle).

Preliminary experiments (not reported here) were conducted to determine the optimal concentration of the sonoelectrochemical NPw per well on Sabouraud dextrose agar seeded with *Candida albicans* after 24 h of incubation at 37 °C. The masses of NPw introduced per well were 2, 5, and 10 mg diluted in 30 µL of Sabouraud medium. Finally, the mass of 2 mg was retained because its corresponding concentration is sufficient to be efficient against *C. albicans* with a correct detection of the growth inhibition zone diameter (IZD). Then, the two other NPws were tested at this same concentration and the results are exhibited in Figure 4.

As seen previously with trypto casein soy plates [35], blue copper ions (Cu^2+^) are implied, whatever the copper-based material, proving oxidation of the NPcs in the liquid. Cu^2+^ ions being more stable than Cu^+^ contained in Cu_2_O, Cu^2+^ ions are generated by the Cu^+^ cuprite oxidation. Therefore, for the three NPws tested, these Cu^2+^ ions are diffusing in the agar, leading to a concentration gradient but also a color gradient. Two main zones were noticeable: one corresponding to the standard growth IZD and one defined as a growth disturbed zone diameter (DZD) where yeasts were partially affected and less numerous. The DZD included an internal concentric zone containing blue Cu^2+^ ions and an external clear concentric one. This DZD was clearly not observed with bleach because bleach, a positive control of the susceptibility of *Candida albicans*, is an uncolored product at the concentration used and creates a homogeneous uncolored gradient in the agar. In any case, the three powders presented a fungicide activity since, in the case of inefficiency, the candida would have grown up to the hole. Table 4 summarizes the values of the IZD and DZD obtained after exposure to each NPw.

As already demonstrated for bactericidal activity [13], 2 mg of sonoelectrochemical Cu_2_O also has an antifungal activity, which is close to that of the commercial Cu. Indeed, for *C. albicans* assays, the mean IZDs obtained after exposure to sonoelectrochemical and commercial Cu_2_O (8.5 ± 0.2 and 8.5 ± 0.5 mm, respectively) were similar (*p* = 0.66) but slightly less important than after exposure to copper (commercial Cu) (10.2 ± 0.4 mm). The DZDs were similar, whatever the NPw tested (*p* > 0.05). The antifungal activity previously described [18,36] is due to the release of copper ions and the production of reactive oxygen species, leading to the disruption of the yeast’s membrane and DNA alterations. Moreover, the yeast resistance to antifungal drugs is mainly due to the formation of hyphae and biofilm. However, Cu_2_O nanoparticles can disrupt the phenotypic change from spherical to filamentous form, reducing the virulence of *C. albicans*. Finally, upon comparing the two cuprite NPws, it seems that the facets on sonoelectrochemical Cu_2_O nanoparticles do not modify their fungicidal behavior against *C. albicans*.

### 2.3. Hemocompatibility

With the exception of the hemolysis assay for which a higher dose (i.e., 500 µg/mL equal to 0.5 g/L) was assayed, 100 µg/mL was the highest sonoelectrochemical Cu_2_O NPw concentration tested for the assessment of hemocompatibility. Tested concentrations were much lower than those used in the antifungal experiments (i.e., 2 mg diluted in 30 µL of Sabouraud medium and equal to 70 g/L) as the NPcs’ diffusion to the blood is limited in the suggested applications (i.e., dental implants and wound dressings).

#### 2.3.1. Hemolysis

Nanomaterials such as amorphous silica [37], platinum [38], and silver [39] can affect red blood cell function, the most abundant cellular component in the bloodstream, which limits the number of potential applications. The absence of hemolysis, the first criterion of hemocompatibility [10], must be therefore verified systematically. The sonoelectrochemical Cu_2_O NPw was consequently tested. For all tested concentrations (0.1, 1, 10, 25, 50, 100, and 500 µg/mL), this NPw showed no hemolysis as defined by ASTM F756 standard practice [40] for the assessment of hemolytic properties of materials. Indeed, all the values of hemolysis are under 1% or under the limit of detection, whatever the tested concentration of NPw. The absence of hemolysis induced by the NPcs is reassuring for their uses in medical applications.

#### 2.3.2. Platelet Aggregation

Platelets play a pivotal role in preventing undesirable blood loss following vascular injury. NPcs affecting hemolysis can also modify platelet aggregation by direct interaction or indirectly due to fibrinogen adsorption onto the NPc surface, which can favor platelet adhesion and initiate thrombogenesis [37,39,41]. More precisely, among the numerous proposed implied mechanisms by which nanomaterial induces platelet toxicity, literature data [9] mentioned an enhancement in the interaction between platelet and endothelial cells [42] and damage of endothelial cells leading to hypercoagulation [43], for example.

**Figure 5:**
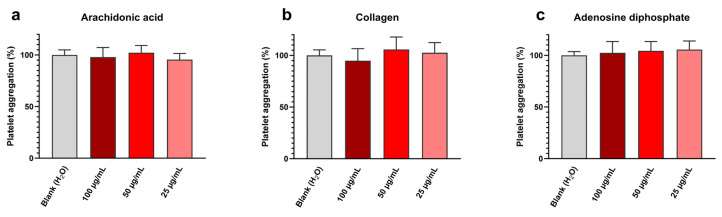
Sonoelectrochemical Cu_2_O nanomaterial does not affect human platelet aggregation, whatever the inducer: aggregation initiated by (**a**) arachidonic acid, (**b**) collagen, and (**c**) adenosine diphosphate. Experiments were performed in duplicate on the blood of three donors. Results are presented as mean ± standard deviation.

Platelet aggregation assay results are shown in Figure 5 and exhibit at all tested concentrations and with all inducers (i.e., arachidonic acid, collagen, and adenosine diphosphate) a platelet aggregation near the blank one. Detailed values are given in Table 5.

Therefore, it can be concluded that synthesized Cu_2_O NPw does not affect platelet aggregation in these conditions. These results are in line with our previously published work [44]. This absence of toxic effects on platelets can be explained by the anionic surface of cuprite sonoelectrochemical NPcs, the zeta potential value previously determined in water being equal to −13 mV [13]. Indeed, due to the zeta potential of platelets being negative, the addition of cationic NPcs, i.e., with a minus superficial charge, reduces the platelet surface charge and, therefore, leads to a bridge between platelets [45]. This charge effect favors platelet aggregation. The experimental results on Cu_2_O NPcs are, therefore, encouraging and indicate that the synthesized NPw can be used in medical applications without promoting platelet aggregation.

#### 2.3.3. Coagulation

The impact of nanomaterials on blood coagulation needs to be assessed as more and more nanoparticles are associated with coagulation disorders [46,47] due to the fact that the activity of coagulation factors may be affected as NPcs are able to modify them [11]. In addition, due to the potential application of sonoelectrochemical NPw as a wound dressing and because coagulation plays a major role in wound healing, the impact of synthesized NPcs on blood coagulation needs to be assessed to avoid prolonged bleeding and delayed wound healing.

**Figure 6:**
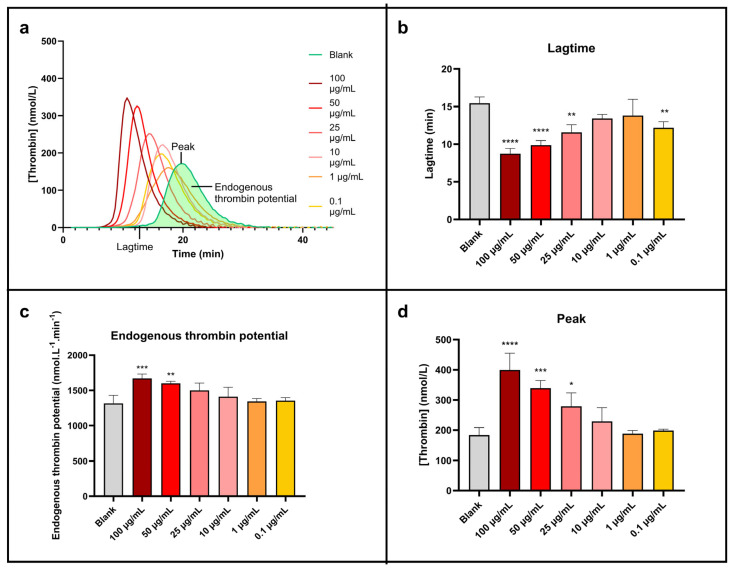
Sonoelectrochemical Cu_2_O nanomaterial facilitates human blood coagulation triggered by exogenous pathways. The impact of nanomaterial on coagulation was assessed by calibrated thrombin generation triggered by MP reagent. Representation of (**a**) thrombin generation curve and thrombin generation parameters displayed as mean ± standard deviation: (**b**) lagtime, (**c**) endogenous thrombin potential, and (**d**) peak. Statistical analyses were performed by one-way analysis of variance with multiple comparisons (versus control). * *p* < 0.05, ** *p* < 0.01, *** *p* < 0.001, and **** *p* < 0.0001.

Thrombin generation test results are exhibited in Figure 6.

Figure 6a, which presents the thrombin generation curves, clearly shows a shift and an increase in the peak height of the curves linked to the concentration of NPcs. This peak is also getting sharper at the same time. So, the higher the concentration of NPcs, the higher the rate of generated thrombin. The NPcs also allow a reduction in the lagtime (time needed to form the first traces of thrombin). These points are more visible on the histogram’s representations (Figure 6b–d) and are quantified in Table 6.

The maximal concentration of thrombin produced (Figure 6d) is roughly doubled compared to water when the concentration of NPcs is maximum (100 µg/mL). In the same conditions, the corresponding lagtime is divided by about two (Figure 6b), and the total concentration of formed thrombin (i.e., endogenous thrombin potential, Figure 6c) increases by around 30%. Although less marked, these effects exist clearly for 10 µg/mL of NPcs: a 24% increase in the peak concentration of thrombin, a 7% increase in the total concentration of thrombin generated, and a 13% reduction in the lagtime. It is, therefore, demonstrated that cuprite sonoelectrochemical NPcs promote human blood coagulation and have a procoagulant effect when coagulation is triggered exogenously. These data are, therefore, a strong asset for applications that involve coagulation needing to be promoted. Indeed, the exogenous pathway is the natural pathway triggered in case of vessel injury. The effect of Cu_2_O NPcs on this pathway can, therefore, be positive for wound closure since the first step in wound healing is the hemostasis phase to prevent important blood loss [48]. Therefore, Cu_2_O NPcs could be beneficial in wound dressings by reducing the coagulation lagtime (i.e., accelerating the coagulation process). However, in the case of functionalized materials such as coated catheters, these NPcs should be used with caution because the implied close contact between blood flow and NPcs can promote undesirable venous thromboembolism via their pro-thrombotic effect [41].

It is noticeable that the results of this work are analogous to previous published work reporting the assessment of the impact of copper sonoelectrochemical NPcs using the same methodology, which indicates similar results (a decreased lagtime and an increased peak concentration of thrombin) [44].

#### 2.3.4. Global Conclusion on Hemocompatibility Results

Overall, the hemocompatibility of the synthesized cuprite NPw is reassuring. It does not induce hemolysis or platelet aggregation but promotes blood coagulation. For these reasons, the applications will probably be limited to devices that are not in direct contact with the blood (such as tubing for extracorporeal circulation) to avoid the occurrence of thrombosis. This coagulation effect could be linked to Cu^2+^ generation in the medium since Klinkajon et al. [49] reported that these ions facilitate coagulation. It will also be interesting to investigate whether sonoelectrochemical NPw influences the global healing process. Indeed, some studies [48,50] report that metallic copper NPcs stimulate dermis epithelialization and angiogenesis. Cu NPcs also promote collagen synthesis and the proliferation of keratinocytes and fibroblasts in animals. Moreover, according to their intrinsic antimicrobial properties, they strongly limit the growth of a biofilm of bacteria. All these characteristics would undoubtedly be an advantage in wound dressings if they always exist for the copper in its oxidized Cu_2_O form.

## 3. Materials and Methods

### 3.1. Nanopowders

Copper (I) oxide NPw (cuprite, Cu_2_O, no. CAS = 1317-39-1) was elaborated by out-of-phase pulsed sonoelectrochemistry. The fact that electrolysis and ultrasound never take place at the same time explains the qualifier “out-of-phase”. The elaboration process used in this work involved three steps per cycle to produce NPcs: 100 ms of potentiostatic electrodeposition followed by 100 ms of ultrasound waves, and then a pause of 100 ms. After many cycles and at the end of the process, NPw was removed from the electrolyte by filtration. After washing with water and ethanol, NPw was dried under a vacuum. Due to the small production scale, the final studied NPw was pooled from different syntheses and was used raw, i.e., without surfactant, for all the analyses and experiments. More details can be found in Reference [13].

Commercial cuprite Cu_2_O and commercial copper (Cu, no. CAS = 7440-50-8) NPws [13] were used for comparison, i.e., Cu_2_O NPw with 18 nm diameter particles (US Research Nanomaterials, Inc., Houston, Texas, USA) and Cu NPw with diameter inferior to 50 nm (Aldrich, Saint-Louis, MO, USA).

### 3.2. Nanopowder Characterization Techniques

Physicochemical analyses of the NPws were carried out as described in detail previously with the same apparatus and with the same technical conditions [13]. Thus, some results were not included in this text when they were very similar to the previous ones (X-ray diffraction patterns, for example).

### 3.3. Antifungal Assays

The antifungal activity of the different nanomaterials was studied using the antibiogram principle and performed on *C. albicans* ATCC^®^ 10231™ provided by American Type Culture Collection (Manassas, VA, USA). First, yeasts were cultured into 10 mL of Sabouraud dextrose broth (Biokar Diagnostics, Beauvais, France) for 24 h at 37 °C in a humidified 5% CO_2_/95% air atmosphere incubator (ThermoFisher Scientific, Illkirch, France). Then, 1 mL of the resulting suspension was added to 9 mL of fresh Sabouraud broth and incubated at 37 °C for 24 h. To isolate colonies, 1 mL of the solution was stripped on a Sabouraud dextrose agar plate (Ø 90 mm) and incubated at 37 °C for 24 h. The final suspension was made by transferring *C. albicans* colonies from Sabouraud dextrose agar to fresh Sabouraud broth and adjusting the concentration to 2 × 10^6^ UFC/mL using a spectrophotometer (V-1200, VWR^®^, Rosny-sous-Bois, France) at 600 nm. Then, 2 mL of the suspension was spread on Sabouraud dextrose agar, and the excess was removed. Four 6 mm diameter wells were drilled and distributed on Sabouraud dextrose agar plates. Three wells were filled with 30 µL of Sabouraud medium, and 2 mg of each of the studied NPws was added to each respective well. The remaining well was used as positive control and filled with 5 µL of bleach containing 2.6% of active chlorine. After 24 h of incubation at 37 °C, the antifungal efficiency was determined using an inhibition zone reader (Scan^®^ 1200, Interscience, Saint-Nom-la-Bretèche, France). The inhibition zone diameter was automatically recorded in mm with a measurement accuracy of ±0.3 mm. This antifungal assay was repeated four independent times.

### 3.4. Hemocompatibility Testing

All hemocompatibility assays were repeated at least three times independently.

#### 3.4.1. Biological Material for Platelet and Coagulation Assessment

Human blood was collected in healthy volunteers in accordance with the Declaration of Helsinki. The sample collection procedure and written informed consent were approved by the Medical Ethical Committee of the CHU UCL Namur (agreement B03920096633). Samples were stored at the NAmur Biobank Exchange (NAB-X, agreement BB1990116).

Whole blood was collected by venipuncture in citrate tubes (BD Vacutainer^®^, Franklin Lakes, NJ, USA) from healthy donors without known coagulation disorders and renal or hepatic chronic disease and free of anticoagulant for at least 2 days. Washed red blood cells (for hemolysis assay) were prepared by centrifuging the whole blood for 5 min at 3000× *g* and washing the pellet three times using the same volume of phosphate-buffered saline as plasma. Platelet-rich plasma (for platelet aggregation assay) was prepared by centrifugation of whole blood at 200× *g* for 10 min at room temperature. Platelet-poor plasma was used as control and prepared by centrifuging platelet-rich plasma at 2000× *g* for 10 min. The platelet count was adjusted at 300,000 platelets per µL. Normal pooled plasma (for coagulation assessment) was prepared by pooling platelet-poor plasma from 41 individuals. The population was composed of 8 men and 33 women, aged from 18 to 57 years (mean ± standard deviation (SD): 25 ± 8 years) with body mass index ranging from 17.3 to 38.9 kg.m^−2^ (mean ± SD: 22.6 ± 3.7 kg.m^−2^). For normal pooled plasma preparation, platelet-poor plasma was obtained by collecting the supernatant of blood tubes after double centrifugation for 15 min at 2000× *g* at room temperature. Plasmas were then pooled, aliquoted, snap-frozen, and stored at −80 °C. Samples were thawed at 37 °C before use.

#### 3.4.2. Hemolysis

Hemolysis was assessed as previously described [44] using the blood of 3 healthy donors. NPw is suspended in water to assay final concentrations ranging from 0.1 to 500 µg/mL. This suspension was incubated with whole blood or washed red blood cells for 1 h at room temperature. Water and triton X-100 at 1% were used as negative and positive controls, respectively. Samples were then centrifuged for 10 min at 2000× *g*. A total of 20 µL of supernatant was incubated for 15 min at room temperature with 180 µL of Drabkin’s reagent, which contains potassium ferricyanide, potassium cyanide, and potassium dihydrogen phosphate as components. This reagent allows the transformation of hemoglobin into methemoglobin and then into cyanmethemoglobin. In other words, it reacts with all forms of hemoglobin except sulfhemoglobin to form cyanmethemoglobin that could be quantified by spectrophotometry. This product level is determined by optical density (OD) at 540 nm using the SpectraMax iD3 (Molecular Devices, San Jose, CA, USA). Results were expressed as hemolysis percentage H (%) using the following equation:

$$H\left(\%\right)=\frac{{\mathrm{OD}}_{540\mathrm{nm}}\mathrm{sample}-{\mathrm{OD}}_{540\mathrm{nm}}\mathrm{water}}{{\mathrm{OD}}_{540\mathrm{nm}}\mathrm{TtitonX}-100-{\mathrm{OD}}_{540\mathrm{nm}}\mathrm{water}}\times 100$$

For each condition, an interference condition was subtracted. The interference condition consists of performing the same assay without whole blood or washed red blood cells. Experiments were performed in triplicate.

#### 3.4.3. Platelet Aggregation

Platelet aggregation was tested by light transmission using the chronometric aggregometer 490-2D. Platelet-rich plasma was incubated with the NPw at concentrations ranging from 25 to 100 µg/mL for 3 min at 37 °C. Platelet aggregation was then induced by three inducers: (a) adenosine diphosphate at 20 µmol·L^−1^, (b) collagen at 10 µg/mL, and (c) arachidonic acid at 1.5 mmol·L^−1^ (Agro-Bio, La Ferté-Saint-Aubin, France). Data were collected with a Chronolog (Havertown, PA, USA) two-channel recorder at 405 nm. Results were expressed in percentage compared to the control (water). All experiments were repeated six times.

#### 3.4.4. Coagulation

The impact of sonoelectrochemical NPw on blood coagulation was assessed using a calibrated thrombin generation test in normal pooled plasma at the different concentrations: 0.1, 1, 10, 25, 50, and 100 µg/mL. Briefly, 80 µL of normal pooled plasma was added to 10 µL of NPcs suspension and to 20 µL of MP reagent (Thrombinoscope BV, Leiden, The Netherlands) that provided 4 µmol·L^−1^ of phospholipids. The assay was also run by substituting the MP reagent with a thrombin calibrator (Thrombinoscope BV) to provide a calibration curve for each condition. The plasma clotting was triggered by the addition of a mix of calcium and a fluorogenic substrate (FluoBuffer^®^ and Fluo substrate^®^, Thrombinoscope BV) at the start of the measurement. The substrate hydrolysis was recorded at 37 °C in a microtiter plate fluorometer (Fluoroscan Ascent, Thermo Labsystems, Helsinki, Finland) using the Thrombinoscope^®^ software v 5.0 (Synapse BV, Maastricht, The Netherlands). The following parameters were extracted from the thrombin generation curves: lagtime (min), peak (nmol·L^−1^), and endogenous thrombin potential (nmol·L^−1^·min). Percentages are expressed in comparison with blank (water). All the experiments were repeated nine times with at least three measurements per second.

### 3.5. Statistical Analyses

Regarding the antifungal assay, the Shapiro–Wilk test, which is the most used to evaluate if the distribution of the samples is normal, was first employed. Indeed, it is suitable for both small and large samples and was carried out using GraphPad Prism (GraphPad Software, v 10.0.2, La Jolla, CA, USA). The obtained *p*-value was less than 0.05, meaning that there was no normal distribution of our data. Therefore, the use of the non-parametric Wilcoxon–Mann–Whitney test was necessary and allowed us to perform the pair-wise comparison of datasets.

For hemocompatibility assays, statistical analyses were carried out by one-way analysis of variance with multiple comparisons to compare each condition with the control using GraphPad Prism Software v 10.1.2.

## 4. Conclusions and Perspectives

Polydisperse and polymorphological cuprite nanoparticles were produced by out-of-phase sonoelectrochemistry, a cheap and environmentally friendly method that offers NPw production in an aqueous medium, limiting chemical products, by-product synthesis, and energy consumption. The so-obtained NPcs could be employed in various applications linked to the oxide properties (catalysis, biocide efficiency…) since ultrafine particles are obtained (92% of particle sizes below 25 nm and around 80% of particle sizes below 10 nm). To our knowledge, this sonoelectrochemical powder is finer than those currently marketed, and this point is essential for strong interactions with the surrounding environment, whatever the application. In the aim of medical applications, antifungal activities on *C. albicans* were investigated. They are comparable to the less versatile copper (reference material) ones from 2 mg diluted in 30 µL of Sabouraud medium on an agar plate. This fungicide behavior of the Cu_2_O NPw adds to antibacterial effects already observed for the same materials at the same concentration. These sonoelectrochemical NPcs could, therefore, be used to prevent nosocomial diseases and infections, whether bacterial or fungal. Hemocompatibility studies were then carried out to ensure the safety of these nanomaterials on wounds. They reveal no hemolysis induction nor platelet aggregation until the high dose of 100 µg of NPw/mL for the three tested aggregation inducers (arachidonic acid, collagen, and adenosine diphosphate). Finally, the calibration thrombin generation tests demonstrate that cuprite sonoelectrochemical NPcs promote human blood coagulation and have a procoagulant effect when coagulation is triggered exogenously. A dose-dependent effect is revealed, i.e., the higher the concentration of NPcs, the higher the concentration of generated thrombin and the total concentration of thrombin, while the corresponding clotting time presents the inverse trend. However, for 10 µg/mL of NPcs, these effects appear clearly (nearly a one-quarter increase in the peak concentration of thrombin, a 7% increase in the total concentration of thrombin generated, and a 13% reduction in the lagtime). Sonoelectrochemical Cu_2_O NPcs could then be used in applications such as wound dressings or operative sheets in hospitals. However, the assays of hemocompatibility are preclinical, and the cytotoxicity of the studied nanoparticles in other human cells (osteoblasts in particular to study the possibility of NPcs inclusion into dental implants or prosthetics) must be evaluated before the marketing of so functionalized medical devices.

## Figures and Tables

**Table 1:** Hydrodynamic diameter for peak values extracted from Figure 2.

Sample of Nanopowder	Diameter Extracted from Relative Number Graph (Figure 2a) (nm)	Diameter Extracted from Relative Weight Graph (Figure 2b) (nm)
Sonoelectrochemical Cu_2_O	7	498
Commercial Cu_2_O	10	28 and 568
Commercial Cu	7	407

**Table 2:** Repartition percentages of particle diameter determined from relative number graph (Figure 2a).

Diameter Range (nm)	Sample of Nanopowder		
	Sonoelectrochemical Cu_2_O	Commercial Cu_2_O	Commercial Cu
0–10	79	10	49
10–15	9	39	20
15–25	6	32	11
25–50	3	14	4
50–100	1	3	2
>100	2	2	14

**Table 3:** Repartition percentages of particle diameter determined from relative weight graph (Figure 2b).

Diameter Range (nm)	Sample of Nanopowder		
	Sonoelectrochemical Cu_2_O	Commercial Cu_2_O	Commercial Cu
<100	≈0	1	≈0
100–250	≈0	2	6
250–500	29	20	61
>500	71	77	33

**Table 4:** Mean inhibition zone diameters and mean disturbed zone diameters of different studied microorganisms after exposure to the three nanopowders tested.

Sample of NPw	MRSA [13]	*E. coli* [13]	*C. albicans*	
	Mean IZD (mm)	Mean IZD (mm)	Mean IZD (mm)	Mean DZD (mm)
Sonoelectrochemical Cu_2_O	14.5 ± 0.9	9.9 ± 0.3	8.5 ± 0.2	28.8 ± 0.3
Commercial Cu_2_O	13.6 ± 0.5	10.5 ± 0.5	8.5 ± 0.5	29.0 ± 0.3
Commercial Cu	14.8 ± 0.6	12.0 ± 0.3	10.2 ± 0.4	29.7 ± 3.3

**Table 5:** Data extracted from Figure 5 concerning the impact of the sonoelectrochemical Cu_2_O nanomaterial on human platelet aggregation initiated by (a) arachidonic acid, (b) collagen, and (c) adenosine diphosphate.

			Platelet Aggregation (% ± Standard Deviation)		
Incubation Medium		Platelet Inducers Tested	Arachidonic Acid (Figure 5a)	Collagen (Figure 5b)	Adenosine Diphosphate (Figure 5c)
Control	Water (blank)		100 ± 5	100 ± 5	100 ± 2
Sonoelectrochemical Cu_2_O nanopowder (concentrations in µg/mL)	100		98 ± 7	95 ± 11	102 ± 9
	50		102 ± 5	106 ± 10	104 ± 8
	25		96 ± 5	103 ± 8	106 ± 8

**Table 6:** Data extracted from Figure 6 concerning the impact of the sonoelectrochemical Cu_2_O nanomaterial on human blood coagulation triggered by the exogenous pathway using MP reagent.

Tested Condition		Lagtime (Figure 6b)		Endogenous Thrombin Potential (Figure 6c)		Peak (Figure 6d)	
		Mean (min ± Standard Deviation)	% ± Standard Deviation	Mean (nmol·L^−1^·min ± Standard Deviation)	% ± Standard Deviation	Mean (nmol·L^−1^ ± Standard Deviation)	% ± Standard Deviation
Control	Water (blank)	15.4 ± 0.9	100 ± 5	1315 ± 117	100 ± 9	184 ± 25	100 ± 13
Sonoelectrochemical Cu_2_O nanopowder (concentrations in µg/mL)	100	8.7 ± 0.7	57 ± 5	1671 ± 61	127 ± 5	399 ± 56	217 ± 30
	50	9.9 ± 0.6	64 ± 4	1600 ± 31	122 ± 2	339 ± 26	184 ± 14
	25	11.6 ± 1.0	75 ± 7	1501 ± 104	114 ± 8	279 ± 45	152 ± 24
	10	13.4 ± 0.6	87 ± 4	1412 ± 133	107 ± 10	229 ± 46	124 ± 25
	1	13.8 ± 2.2	90 ± 14	1344 ± 42	102 ± 3	188 ± 11	102 ± 6
	0.1	12.2 ± 0.9	79 ± 5	1355 ± 44	103 ± 3	199 ± 5	108 ± 2

## Data Availability

The original contributions presented in this study are included in this article; further inquiries can be directed to the corresponding author(s).
